# Single Domain Antibody application in bacterial infection diagnosis and neutralization

**DOI:** 10.3389/fimmu.2022.1014377

**Published:** 2022-09-29

**Authors:** Qian Qin, Hao Liu, Wenbo He, Yucheng Guo, Jiaxin Zhang, Junjun She, Fang Zheng, Sicai Zhang, Serge Muyldermans, Yurong Wen

**Affiliations:** ^1^ Department of General Surgery, Center for Microbiome Research of Med-X Institute, The First Affiliated Hospital, Xi’an Jiaotong University, Xi’an, China; ^2^ The Key Laboratory of Environment and Genes Related to Disease of Ministry of Education, Health Science Center, Xi’an Jiaotong University, Xi’an, China; ^3^ Center for Biomedical Research, Institute of Future Agriculture, Northwest A&F University, Yangling, China; ^4^ Laboratory of Cellular and Molecular Immunology, Vrije Universiteit Brussel, Brussels, Belgium

**Keywords:** single domain antibody, nanobody, bacterial infection, diagnosis, neutralization

## Abstract

Increasing antibiotic resistance to bacterial infections causes a serious threat to human health. Efficient detection and treatment strategies are the keys to preventing and reducing bacterial infections. Due to the high affinity and antigen specificity, antibodies have become an important tool for diagnosis and treatment of various human diseases. In addition to conventional antibodies, a unique class of “heavy-chain-only” antibodies (HCAbs) were found in the serum of camelids and sharks. HCAbs binds to the antigen through only one variable domain Referred to as VHH (variable domain of the heavy chain of HCAbs). The recombinant format of the VHH is also called single domain antibody (sdAb) or nanobody (Nb). Sharks might also have an ancestor HCAb from where SdAbs or V-NAR might be engineered. Compared with traditional Abs, Nbs have several outstanding properties such as small size, high stability, strong antigen-binding affinity, high solubility and low immunogenicity. Furthermore, they are expressed at low cost in microorganisms and amenable to engineering. These superior properties make Nbs a highly desired alternative to conventional antibodies, which are extensively employed in structural biology, unravelling biochemical mechanisms, molecular imaging, diagnosis and treatment of diseases. In this review, we summarized recent progress of nanobody-based approaches in diagnosis and neutralization of bacterial infection and further discussed the challenges of Nbs in these fields.

## Introduction

With the increasing antibiotic resistance, bacterial infection constitutes a serious threat to human health. It can lead to tremendous morbidity and mortality, emphasizing the need for rapid and effective identification and treatment of bacteria pathogens (1). At present, clinical bacterial diagnosis mainly involves bacterial culture, molecular diagnostics and colony formation methods which are time-consuming, labor intensive and requiring expensive equipment, all of which limit the utility, especially in resource limited settings (2–4). Oral and intravenous antibiotics are the most common treatments against bacterial infections; however, they are usually administered against ill-defined pathogens. This abuse of antibiotics plays an important role in the increase of antibiotic resistance (5–8). Therefore, it is important to develop fast, cost-effective, and accurate methods for the detection, identification and treatment of bacterial infections. Antibodies became promising molecules for bacterial detection and treatment due to their high sensitivity and specificity.

Antibodies are essential components of adaptive immunity. Antibody-based diagnosis and therapeutics are the fastest growing classes of drugs on the market. The US FDA has approved over 100 antibodies mainly for treating cancer (45%) and immune-mediated disorders (27%) while only 8% against infectious diseases (9). The high production cost, low stability and large size may be the main obstacles to develop the antibodies for treating infectious diseases (10). Therefore, single domain antibodies (sdAbs), which have low production costs, high stability and small size become a promising alternative to canonical antibodies (11).

**Figure 1:**
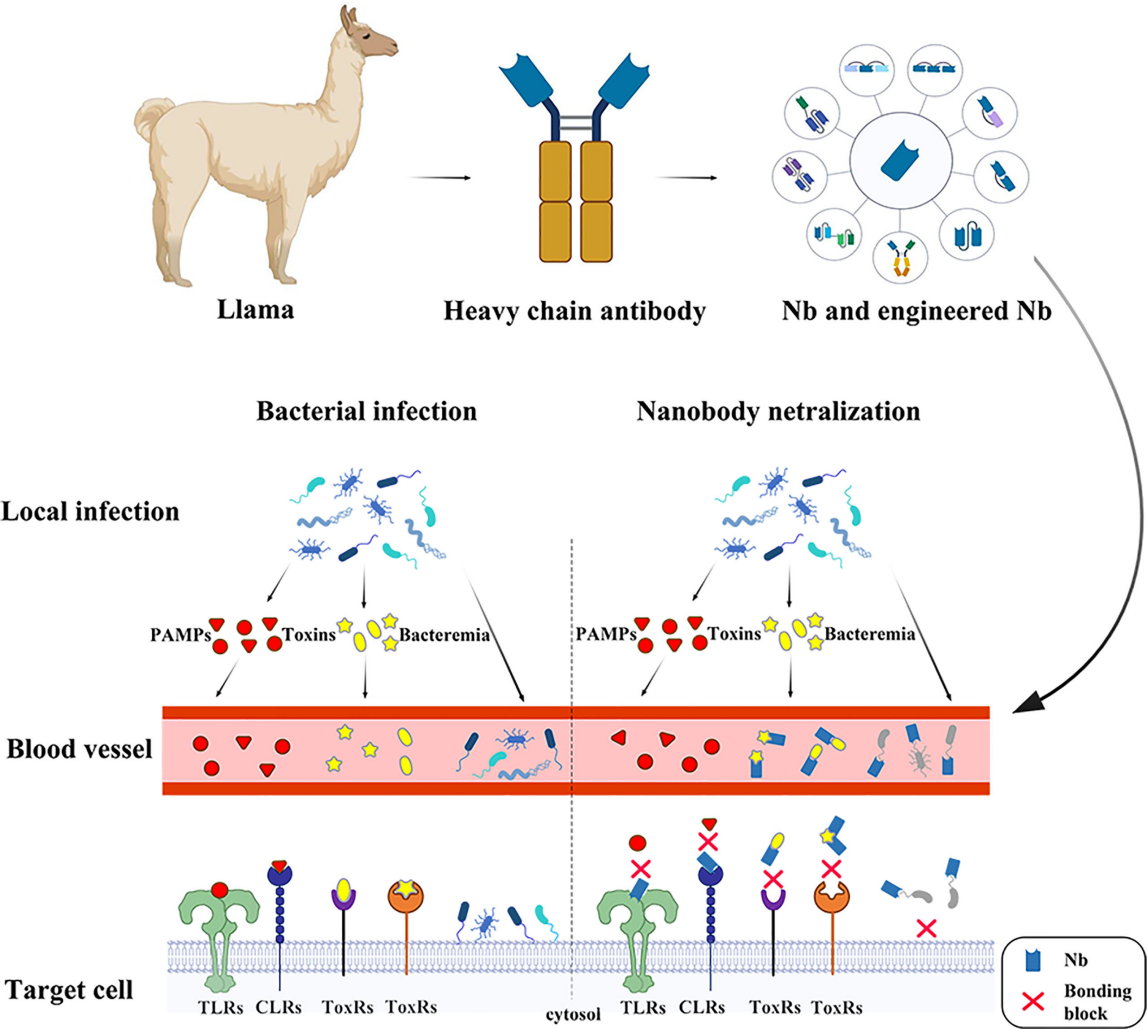
The nanobodies take effects in several ways against bacterial infection. In the early stage of infection, pathogenic microorganisms are confined to the lesion. At this time, the PAMPs and the toxins are released into the bloodstream. The nanobodies binding onto the receptors prevent PAMPs recognition by PRRs, such as Toll-like receptors, Nod-like receptors and C-type lectins such as Clec4f, leading to a series of bodily reactions. The toxins, such as CDT, Tcd and BoNT are the major part in bacterial pathogenicity, which serve in different ways to cause damage in hosts and assist in enlargement of infection foci. The neutralizing nanobodies protect the host through specifically binding the toxins. At late stage of infection, pathogens are released into blood causing bacteremia. The nanobodies recognizing surface antigens, such as pilus and flagellum, bind onto the pathogen surface, preventing bacterial attachment.

In 1990s, scientists found a unique class of “heavy-chain-only” antibodies (HCAbs) in the serum of camelids and sharks. Owing to the absence of light chains, HCAbs binds to the antigen through only one variable region, referred to as VHH or also sdAb or nanobody (Nbs). The antigen-binding domain of shark HCAbs are known as VNAR (12, 13). Their special structure endowed sdAbs with superior properties and enabled them to be extensively employed in structural biology (14–16), unravelling biochemical mechanisms (17), molecular imaging (18–20), diagnosis and treatment of tumors (21, 22) and infection diseases (23–27). As for infectious diseases, sdAb have been widely used in the diagnosis and treatment of a variety of viral infections (28). It is noteworthy that a lot of nanobodies have been generated targeting the severe acute respiratory syndrome coronavirus 2 (SARS-CoV-2, COVID-19), and there have been excellent reviews to summarize the current research progress (29, 30). This review will focus on the current progress and perspectives of diagnostic and neutralizing sdAbs against bacterial infection (**Figure 1**).

## Structural and physicochemical features of Single Domain Antibodies

SdAbs are the smallest known natural antigen-specific binding functional fragment, with dimensions of 2.5 nm in diameter and 4 nm in length. They consist of approximately 120 amino acids and merely 12~17 kD in weight, which is only one-tenth of canonical antibodies (150kD) (31). Similar to the VH domain of canonical antibodies, sdAbs consist of three hypervariable antigen-binding loops (complementarity determining regions, CDR1-CDR3) and four conserved framework regions (FR1-FR4) (32–34). There are mainly two differences between sdAbs and VHs of canonical antibodies. SdAbs have elongated CDR1 and CDR3, which to some extent compensate the loss of antigen-binding surface contributed by the light chain CDRs. In addition, the elongated CDR3 can adopt larger variety of structures and has a preference to interact with concave shaped antigen surfaces (35). Another notable difference is the conservative hydrophobic amino acids (Val47, Gly49, Leu50, and Trp52) in canonical antibody FR2 substitution by hydrophilic amino acids (Phe42, Glu49, Arg50, and Gly52), increasing solubility and stability of sdAbs (33, 36).

Due to the specific structure, sdAbs possess several outstanding characteristics compared to the traditional antibodies. 1) SdAbs represent the smallest naturally derived antigen-binding functional fragments (~15 kD). The small size allows sdAbs to penetrate deeper in dense tissues and might cross the blood-brain barrier(BBB) (37, 38), and to be quickly eliminated *via* the kidney (39). Besides, the higher isoelectric point makes SdAbs positively charged and easier to penetrate the BBB. Therefore, sdAbs are more suitable for targeting solid tumors (40, 41) and brain diseases (37, 42, 43). 2) Compared with traditional antibodies, the sdAbs have only one domain with disulfide bonds, which folds into a relatively stable structure. Amazingly, sdAbs maintained their antigenic binding ability after being incubated for one week at 37°C. They even can tolerate higher temperatures of 60-80°C (36). In some cases, they can regain antigen-binding activity after thermal denaturation by exposure at high temperatures (90°C) (44).When exposed to chemical denaturing agents and proteases, as well as non-physiological pH (pH range 3.0-9.0), sdAbs can also retain most antigen-binding capabilities (45). These properties permit the use of more demanding chemical and physical conditions during treatments or modifications of sdAbs than other types of antibodies. 3) The longer amino acid sequences of the CDR3 enlarges the antigen binding surface of the sdAbs, increases their structural repertoire, and further expands the binding ability to some hidden antigenic epitopes by creating new finger-like structures. Thus, they are enhancing the recognition ability of concave epitopes as well as binding to such epitope architectures with high affinity (31, 46–51). 4) The hydrophilic amino acids on the side of VHH, corresponding to the VL interface of VH domains, improve the solubility in aqueous solutions and lower the tendency to aggregate (52). 5) The high degree of sequence identity with human VH domains of family-3 of VHHs, their small size, resistance to form aggregates and rapid blood clearance favor a low immunogenicity. VNARs may have a higher immunogenicity due to low sequence identity between VNARs and human VH or VL domains (~ 30% overall) (53). Overall, the immunogenicity risks with VHHs are low (54). In addition, the humanization of sdAbs provides a safe option for long-term treatment (55, 56). 6) SdAbs can be efficiently, easily and economically produced recombinantly in bacteria, mammalian cell lines, yeast and plants at an affordable cost (11, 57), while the production of canonical monoclonal antibodies requires mammalian expression system, which is complex in technology and expensive to maintain. Apart from these outstanding characteristics, sdAbs also have some limitations. The biggest drawback of sdAbs is their inadequate pharmacokinetics. Compared with conventional antibodies, sdAbs have a faster serum clearance rate, which limits their application in the field of therapy. In addition, sdAbs may have some adverse effects and a humanized tetravalent Nb have been reported with hepatotoxicity. The characteristics compared between nanobodies and conventional antibodies are listed in **Table 1**.

**Table 1:** Characteristics compared between nanobodies and conventional antibodies.

Characteristics	Nanobodies	Conventional antibodies
The molecular weight	Low (~15 kDa)	High (~150 kDa)
Stability	High	Low
Affinity	High	Low
Solubility	High	Low
Immunogenicity	Low	High
Cost	Economic	Expensive
Serum clearance rate	Fast	Slow

## Single Domain Antibody use to diagnose and neutralize infections by Gram- negative bacteria

### Enterotoxigenic *E. coli*

*Enterotoxigenic E. coli* (ETEC) is one of the most common causes of diarrhea in toddlers, adults in the developing world and in travelers to endemic areas. According to WHO reports, ETEC related diarrhea is one of the leading causes of death in the children under the age of 5 in developing countries (58). In addition, ETEC strains causing severe, watery diarrhea are responsible for significant death and morbidity in neonatal and post-weaned piglets, leading to worldwide tremendous economic losses in pork industry (59).

ETEC is a non-invasive pathogen that mediates small intestine adherence through bacterial surface structures, known as colonization factors (CFs). Once bound to the small intestine, the bacteria produce toxins causing a net flow of water from enterocytes, leading to watery diarrhea (60). ETEC strains can also produce many types of fimbriae that are involved in bacterial attachment. F4 fimbriae are commonly found on ETEC from diarrheic piglets (61, 62). In 2005, Harmsen et al. immunized a llama with F4ac fimbriae from the F4-positive (F4+) ETEC strain CVI-1000 and obtained a few monoclonal VHHs. However, the best monovalent VHH, K609, could not significantly reduce diarrhea and reduced piglet mortality was poor (63). In contrast, orally administration of the linker connected bivalent VHH could enhance the clearance of F4+ ETEC and decrease the number of infected piglets (64). The different *in vivo* activity of mono- and bivalent VHH suggested that the ability to agglutinate bacteria may have a higher impact on infection, consistent with other studies where only bivalent antibodies showed *in vivo* protection (65–67). In another study, Moonens et al. (59) fused four different variable domains of llama heavy chain-only antibodies (V1-4), raised against F4ac, to the Fc domain of a porcine immunoglobulin IgA. These four different VHH targeted conserved epitopes of FaeG, a major adhesive subunit of F4. The four VHHs were fused to porcine IgA-Fc and subsequently expressed in *Arabidopsis thaliana* seeds to feed piglets. The oral feed-based passive immunization strategy protected piglets as demonstrated by (i) the progressive decline in shedding of F4 positive ETEC bacteria, (ii) the significantly lower immune responses of the piglets to F4 fimbriae, which suggest a reduced exposure to the ETEC pathogen, and (iii) a significantly higher body weight in comparison with control piglets (63, 68). The structural study of V1-4 in complex with FaeG indicated that they sterically hindered FaeG associating with the F4 receptor but they did not directly interfere with the carbohydrate binding site (59). Besides F4+ ETEC, four VHHs targeting F18 fimbriae FedF domain were generated by llama immunization and selection as well. They could inhibit F18+ ETEC attaching to piglet enterocytes *in vitro*, and either sterically hinder or induce conformational changes of the binding surface of FedF (69). In a recent study, Amcheslavsky (60) and his colleagues immunized two male llamas with N-terminal fragments of eight class-5 ETEC adhesins to generate nanobodies with broad cross-reactivity against ETEC adhesins. They identified single nanobodies that show cross protective potency against eleven major pathogenic ETEC strains *in vitro* and inhibited ETEC colonization *in vivo*. Molecular docking and mutagenesis analysis revealed that nanobodies recognized a highly conserved epitope within the putative receptor binding region of ETEC adhesins (60).

### Shiga toxin-producing Escherichia coli

*Shiga toxin-producing Escherichia coli* (STEC) are a subset of *E. coli* pathogens leading to illnesses such as diarrhea, hemolytic uremic syndrome (HUS) and even death. Shiga toxins, the main virulence factors are divided in two groups: Stx1 and Stx2, of which the latter is more frequently associated with severe pathologies in humans and newly weaned pigs (70). Stx2e consists of an enzymatically active A subunit and five B subunits that bind to globotriaosylceramide (Gb3) on host cells (71). Lo et al. reported the discovery and characterization of a VHH, NbStx2e1, isolated from a llama phage display library that confers potent neutralizing capacity against Stx2e toxin. Structural analysis revealed that for each B subunit of Stx2e, one NbStx2e1 is interacting in a head-to-head orientation and directly competing with the glycolipid receptor binding site on the surface of the B subunit. The neutralizing NbStx2e1 can be used to prevent or treat edema disease in the future. Tremblay et al. immunized llama with Stx1 and 2 together and identified a panel of neutralizing VHHs, two of which demonstrated cross activity to Stx1 and 2 (72). A VHH heterodimer consisting of one Stx1-specific VHH/Stx2-specific VHH, and one Stx1/Stx2 cross-specific VHH, significantly improved the survival and reduced the kidney damage of mice challenged with Stx1 or 2. In addition, co-administration of the heterodimeric VHH with an effector Ab that binds to the VHH heterodimer, was effective in preventing all symptoms of intoxication from Stx1 and Stx2. In 2016, Mejías and his colleagues reported the generation of a family of Stx2B-binding VHHs that neutralize Stx2 *in vitro* at a nanomolar to subnanomolar range. The anti-Stx2B VHH, 2vb27, was selected and two copies were fused to an anti-human serum albumin VHH. This engineered antibody showed increased retention in circulation and was able to neutralize Stx2 in three different mouse models. This novel and simple antitoxin agent should offer new therapeutic options for treating STEC infections to prevent or ameliorate HUS outcome (73). In another study, Navarro et al. described the identification and characterization of a nanobody (Nb113) with the potential to neutralize the Stx2a and Stx2c toxins that are associated with human clinical infections. The crystal structural study revealed that each B subunit in the pentameric B5 ring is associated with a single Nb113 molecule. A detailed analysis of the epitope targeted by Nb113 suggests that this Nb prevents the formation of the Stx2a–Gb3 complex, thereby impeding the subsequent steps of the internalization and enzymatic activity of the Stx2a holotoxin (70).

Besides Stx2-neutralizing VHHs, two VHHs were identified from immunized llama for detection of Stx2 using ELISA, which was even more sensitive than commercial ELISA kits (74). The ELISA was best for the major subtype Stx2a and less sensitive for Stx2f. VHH based ELISA is expected to be more cost effective than IgG ELISA.

### Other Gram- negative bacteria

*Pseudomonas aeruginosa* is one of the leading causes of hospital-acquired infections. It is difficult to treat the infections due to the high intrinsic antibiotic resistance and the organism’s capability to occur in biofilms in the host. Adams et al. immunized a llama with *P. aeruginosa* antigens and identified monoclonal anti-flagellin VHHs. In an *in vitro* assay, they showed that the anti-flagellin VHHs are capable of inhibiting *P. aeruginosa* from swimming and that they prevented biofilm formation (75).

*Helicobacter pylori* infection is associated with gastritis, gastric and duodenal ulcers, and even gastric adenocarcinoma. It is important to seek alternative therapeutic strategies due to the increasing occurrence of antibiotic resistance. Some studies reported the isolation and purification of nanobodies with high affinity against UreC subunit of urease enzyme from *H. pylori*. These nanobodies could be a novel class of treatments against *H. pylori* infection (76, 77). The sdAbs employed for diagnosis and neutralization of Gram- negative bacterial infection are listed in **Table 2**.

**Table 2:** SdAb reports to diagnose and/or neutralizing infections by Gram-negative bacteria.

Nanobody	Source	Target	Structure	(IC50)/KD	Function	Diagnosis/Neutralizing	Ref.
K609	Immune library	ETEC F4 fimbriae	–	–	prevented F4+ ETEC attachment	Neutralizing	(63)
V1 V2 V3	–	ETEC F4 FaeG	4WEM 4WEN 4WEU	0.1 to 7.7 µM	prevent F4+ ETEC attachment	Neutralizing	(59)
NbFedF6 NbFedF7 NbFedF9	Immune library	ETEC F18 FedF	4W6W 4W6X 4W6Y	–	inhibit F18+ ETEC attachment	Neutralizing	(69)
2R215 2R23	naive library	ETEC CfaE	–	0.4125 to 13.3 µM(IC100)	broad cross-protection against 11 major disease causing ETEC strains and prevented colonization *in vivo*	Neutralizing	(60)
1D7 1H4	Immune library	ETEC CfaE	–	–	prevented bacterial colonization in animals.	Neutralizing	(60)
NbStx2e1	Immune library	STEC Stx2e	4P2C	8 nM	direct interaction with the Stx2e B subunit binding site for glycolipid, thereby impeding toxin-host cell receptor contacts	Neutralizing	(71)
2VB27	Immune library	STEC Stx2B			neutralized Stx2 *in vitro* at subnanomolar concentrations	Neutralizing	(73)
Nb113	Immune library	STEC rStx2aB	6FE4	9.6 nM	neutralized Stx2a by competing for the Gb3 receptor	Neutralizing	(70)
Stx-A4 Stx-A5	Immune library	STEC Stx1/Stx2	–	7.2-12.5 nM	neutralized Stx1 and Stx2 and prevented all symptoms of intoxication from Stx1 and Stx2	Neutralizing	(72)
1vb1- 2vb10 2vb21-2vb10	Immune library	STEC Stx2	–	–	early detection of STEC infections	Diagnosis	(74)
7G 9D	Immune library	P. aeruginosa flagellum	–	2.5 nM 4.7 nM	inhibit P. aeruginosa from swimming and prevent biofilm formation *in vitro*	Neutralizing	(75)
nanobody against UreC	Immune library	UreC	–	0.05nM	bind to UreC and inhibit urease activity	Neutralizing	(76)
HMR23	Immune library	UreC	–	0.0263nM	bind to UreC and inhibit urease activity	Neutralizing	(77)

## Single Domain Antibody usage for diagnosis and neutralization of Gram-positive bacterial infection

### Clostridium difficile

*Clostridium difficile* is an opportunistic pathogen residing in the gastrointestinal tract of humans, causing antibiotic-associated diarrhea and pseudomembranous colitis (78). Antibiotics metronidazole and/or vancomycin are the primary treatment for *C. difficile*-associated disease (CDI) and surgeries are often required in the case of fulminant CDI (79). Due to the difficulties of treatment and high rates of recurrence, it’s necessary to explore new therapeutic agents (80). The Gram-positive bacterium produces two large clostridial exotoxins, toxin A (TcdA) and toxin B (TcdB), which are the major virulence factors responsible for CDI and are potential targets for CDI therapy (81). TcdA and TcdB are homologous to each other, having a similar domain organization including glucosyltransferase domain (GTD), cysteine protease domain (CPD), delivery and receptor binding domain (RBD) and combined repetitive oligopeptide domain (CROPs) (82, 83). In 2011, Hussack and his colleagues isolated after phage display from an immune sdAb llama library four VHHs specifically targeting partial CROPs of TcdA or TcdB. *In vitro* assay on fibroblast cells demonstrated potent protection from the cytopathic effects of toxin A by these VHHs. Moreover, the protection efficiency was further enhanced when VHHs were administered in a manner of paired or triplet combinations (81). In another study, they characterized a panel of VHHs against partial RBD and CROPs of TcdB. Unfortunately, none of these VHHs exhibited inhibitory effects against TcdB cytotoxicity in a cell-based assay, given that several VHHs showed high affinity to toxin. This incapability of neutralization is probably due to TcdB accepting multiple proteins as receptors (84–86) and blockage of a single epitope might not be effective inhibition of TcdB toxicity. Nevertheless, when bivalent VHHs fused to the Fc fragment, their neutralization efficiency reached to the level of the recently approved anti-toxin B monoclonal antibody, bezlotoxumab (87). Furthermore, VHHs targeting different vulnerable regions on TcdB were also developed. SdAb named E3, 7F and 5D were demonstrated to bind with GTD, the connecting region between GTD and CPD, and RBD, respectively. Among which, E3 showed the best inhibition of TcdB cytotoxicity (88, 89). Yang and his colleagues created a tetravalent and bispecific antibody called “ABA” which comprised two VHHs against both, TcdA and TcdB. ABA was capable of binding to both toxins simultaneously and neutralizing toxins from clinical *C. difficile isolates*. Therefore, ABA showed a significantly enhanced neutralizing activity both *in vitro* and *in vivo* (90). Schimdt and colleagues constructed a heteromultimeric VHH-based neutralizing agent, which potently neutralized both *C.difficile* toxins in cell assays and protected animals from CDI to different extents (88). In addition to development of VHHs, strategies to administer VHHs were also explored. For example, adenovirus, engineered Lactobacillus and probiotic Saccharomyces boulardii, expressing different forms of VHHs, were utilized to treat CDI effectively in animal models and proved to be promiscuous for combating the diseases invoked by *C. difficile* (91–93). Beside TcdA and TcdB, surface layer proteins (SLPs), mediating adherence to host cells, represents an alternative target for CDI treatment. Kandalaft and his colleagues used SLPs isolated from *C. difficile* hypervirulent strain QCD32g58 (027 ribotype) to immunize a llama and identified a panel of SLP-specific VHHs, which exhibited inhibition of *C. difficile* QCD32g58 motility *in vitro*. Therefore, targeting SLPs with VHHs may be a viable therapeutic approach against CDI (94).

### Bacillus anthracis

Anthrax is a severe and fatal disease caused by the Gram-positive *Bacillus anthracis*. Anthrax toxin is a mixture of one non-toxic protein, protective antigen (PA) and two toxins, edema factor (EF) and lethal factor (LF). Protective antigen (PA) could bind to anthrax toxin receptors on cell surface forming oligomer pore and translocate the lethal factor (LF) and edema factor (EF) into the cytosol to take effects (95). In 2015, Moayeri and his colleagues identified two classes VHHs (JIK-B8 and JKH-C7) targeting two epitopes of PA from immunized alpacas. The two VHHs were expressed as a heterodimeric VHH-based neutralizing agent (VNA2-PA) and displayed improved neutralizing potency in *in vitro* and *in vivo* assays compared with monomeric VHH (96). In another study, they used a gene therapy approach using recombinant replication-incompetent human adenovirus serotype 5 (Ad5) vector to express and secret the VNA (Ad/VNA2-PA) into the serum, and found that it can protect mice against an anthrax toxin challenge and anthrax spore infection (97). Apart from PA, the same group identified a set of 15 VHHs against EF and/or LF. Six of these VHHs were cross-reactive with both, EF and LF N-terminal domain, which is responsible for association with PA. Unlike the other selected VHHs, one LF specific VHH bound the C-terminal of LF inhibiting its enzymatic activity. Two bispecific heterodimers of the selected neutralizing VHHs demonstrated full protection against lethal anthrax spore infection (98).

The cell surface of *B. anthracis* is covered by a protective surface layer or S-layer, composed of the highly-conserved S-layer protein (Sap). S-layers are proposed to function (i) as exoskeletons, (ii) as protection against harmful environments, (iii) as scaffolding structures for surface-localized enzymes and adhesins, (iv) as molecular sieves for nutrient uptake and (v) as a contact zone with the extracellular environment, including host cells in case of pathogenic bacteria (99). Fioravanti et al. generated Sap self-assembly inhibiting nanobodies, which exhibited disruption of the S-layer and attenuated the bacterial growth. Subcutaneous injection of the Sap inhibiting nanobodies cleared anthrax infection and prevented death in a mouse model of anthrax (100).

### Clostridium Botulinum

Botulinum neurotoxins (BoNTs) are a category of bacterial toxins produced by *Clostridium Botulinum* and related strains, they are dangerous potential bioterrorism agents (Category A and Tier 1 select agent) (101). BoNTs cause a life-threatening disease called botulism, which develops flaccid paralysis and autonomic dysfunctions. Once infected, patients have to stay in the intensive care unit (ICU) and rely on mechanical ventilation for weeks to months, which is costly and time consuming (102). There are seven known serotypes of BoNTs (BoNT/A to BoNT/G), in which serotypes A, B and E are often associated with human botulism (103). Currently, antitoxins such as equine antitoxin and human botulism immunoglobulin represent the main strategy for treatment. However, adverse reactions, including early anaphylactic shock and late serum sickness, have been reported (103), which poses the necessity for developing new therapeutics to treat botulism. To this end, nanobodies could play an important role in such tasks.

For this purpose, a variety of VHHs against BoNT/A were generated in the past years from phage or yeast display libraries derived from camel, alpaca and llama, respectively. Thanongsaksrikul et al. reported a neutralizing nanobody, VHH17, binding specifically to the catalytic cleft in light chain of BoNT/A *via* its CDR2 region, which is inaccessible to conventional antibodies due to their large size (104). In a similar study, Dong et al. identified a VHH Aa1 using yeast display. Rather than binding to the catalytic site of BoNT/A, Aa1 targeted the non-catalytic α-exosite binding region and inhibited enzyme activity of the toxin. Besides, Aa1 exhibited extraordinary thermal and reducing stability, which is optimal for therapeutic purposes (105). Tremblay and colleagues identified and characterized two VHHs ALc-B8 and ALc-H7 having affinity up to the nanomolar level to the light chain of BoNT/A. They further confirmed that ALc-B8 was able to inhibit SNAP-25 proteolysis in neuronal cells intoxicated by BoNT/A (106), which demonstrated its potential for therapy. In a recent study, Lam et al. discussed the inhibitory mechanism of VHHs against BoNT/A light chain *via* structural studies and found that the recognized epitopes of the light chain are quite conserved across different subtypes, laying the foundation for structure-based drug design (107, 108). Besides the protease domain, VHHs such as ciA-C2, specifically recognizing the receptor binding domain of BoNT/A were also identified and proven to exert an inhibitory function (109). Furthermore, various strategies to enhance the efficacy of VHHs neutralization of BoNT/A have been exploited, such as (i) tagging the VHHs for better and faster clearance of bound toxin (110), (ii) fusing the VHHs with human Fc fragment or Glycophorin A on red blood cell surface to increase their circulation half-life (111, 112), or (iii) expressing VHHs in replication-incompetent adenovirus to provide prolonged protection (113). With similar strategies, several VHHs bound to BoNT/E were also produced and characterized. Bakherad et al. selected a VHH, BMR2, specifically targeting the receptor binding domain of BoNT/E, which completely neutralized 3LD_50_ of BoNT/E in mice (103). Lately, Tremblay et al. identified plenty of BoNT/E-neutralizing VHHs and Lam et al. characterized two of them, JLE-E5 and JLE-E9, targeting the translocation domain of BoNT/E. They confirmed that these two VHHs blocked a structural change of BoNT/E in acidic pH, a process necessary for its biological function, which could hamper toxicity of BoNT/E (114). The pitfall to treat botulism is that no drugs are able entering into neurons to take effect once the toxins are endocytosed. A hallmark application of VHH for treating botulism was to deliver VHHs into neural cells by coupling them to intoxicated BoNTs. Utilizing this strategy, two independent groups successfully delivered VHHs into neurons and provided animals with full recovery from botulism, which opened new avenues of using VHHs to treat diseases (115, 116).

### Other Gram-positive bacteria

In addition to the bacteria mentioned above, nanobodies also play an important role in the diagnosis and therapy of other bacteria. Nanobodies can also be used to establish immuno-assays to uncover bacteria contaminations in foods. *Staphylococcus aureus* is one of the most common food-borne pathogens. Hu et al. selected a specific nanobody Nb147 to develop an immuno-assay detecting *S. aureus* in milk (117). *Staphylococcal* enterotoxins (SEs) are the major causes of staphylococcal food poisoning (SFP) and various other diseases. Ji et al. developed a double nanobody-based sandwich immunoassay for the detection of staphylococcal enterotoxin C in dairy products (118) while Zanganeh et al. developed a rapid and sensitive detection of staphylococcal enterotoxin B by recombinant nanobodies (119). *Listeria monocytogenes* (LM) causes listeriosis, a potentially fatal food-borne disease especially harmful to pregnant women. Tu and his colleagues developed an ELISA using the VHH clone L5-79 and a monoclonal antibody to detect LM in pasteurized milk (120). King et al. identified a group of VHHs targeting internalin B (InlB) of LM which were competitive inhibitors preventing bacterial invasion. These results point to the potential of VHH as a novel class of therapeutics for the prevention of listeriosis (121). The sdAbs applications to diagnose and neutralize Gram- positive bacterial infection are overviewed in **Table 3**.

**Table 3:** SdAb reports to diagnosis and neutralization of infection by Gram-positive bacteria.

Nanobody	Source	Target	Structure	(IC50)/KD	Function	Diagnosis/Neutralizing	Ref.
A4.2 A5.1 A20.1 A26.8	Immune library	CD TcdA	–	–	neutralized toxin A by binding to sites other than the carbohydrate binding pocket of the toxin	Neutralizing	(81)
B39 B69 B71 B74 B94 B131 B167	Immune library	CD TcdB	–	–	neutralized toxin B when formatted as bivalent VHH-Fc fusions	Neutralizing	(87)
5D,E3,7F	Immune library	CD TcdB	6oQ6 6oQ7 6oQ8	–	neutralized toxin B	Neutralizing	(89)
ABA	Immune library	CD TcdA TcdB	–	–	bound to both toxins simultaneously and displayed a significantly enhanced neutralizing activity both *in vitro* and *in vivo*	Neutralizing	(90)
SLP-VHH	Immune library	CD-SLP	–	–	bound SLPs with high affinity bloking the adherence to host cells	Neutralizing	(94)
VNA2-PA	Immune library	Bacillus anthracis PA	–	–	displayed improved neutralizing potency *in vitro* and *in vivo* than the separate component VHHs	Neutralizing	(96) (97)
JMN-D10 JMO-G1	Immune library	Bacillus anthracis EF/LF	–	–	block binding of EF/LF to the protective antigen C-terminal binding interface and preventing toxin entry into the cell	Neutralizing	(98)
Nbs-NbAF684 nbaf694	Immune library	Bacillus anthracis SAP	–	–	prevented the assembly of Sap and depolymerized existing Sap S-layers	Neutralizing	(100)
VHH17	naive library	BoNTs BoTxA/ LC	–	11.6nm	neutralized the SNAP25 hydrolytic activity of BoTxA/LC	Neutralizing	(104)
BMR2	Immune library	BONT/E HC	–	–	neutralized BoNT/E	Neutralizing	(103)
Aa1	naive library	BONT/A-LC	3K3Q	4.7×10^-10^M	targeted the non-catalytic α-exosite binding region and inhibited enzyme activity of toxin	Neutralizing	(105)
ALc-B8 ALc-H7	Immune library	BONT/A-LC	–	–	neutralized BoNT/A-LC and inhibit SNAP-25 proteolysis in neuronal cells	Neutralizing	(106)
JLK-G12 JLO-G11 JLI-G10 JLI-H11	Immune library	BONT/B-HC	6UFT 6UL4 6UHT 6UC6	–	block BoNT/B1 binding to host receptors	Neutralizing	(108)
ciA-B5 ciA-H7 ciA-C2	Immune library	BONT/A1- HN LC HC	6UL6 6UI1 5L21	–	block membrane insertion of boNT/A1 translocation domain, interfere with the unfolding of the protease domain, block host receptor binding	Neutralizing	(108, 109)
B11 G3	Immune library	BoNT/A	–	–	neutralized BoNT/A	Neutralizing	(111)
H7/B5/ABP	Immune library	BoNT/A	–	<3 nM	neutralized BoNT/A	Neutralizing	(122) (113) (112)
JLE-E5 JLE-E9	Immune library	BoNT/E1	7K84 7K7Y	–	block membrane association of BoNT/E1	Neutralizing	(114)
A8-J10-ciBoNT/XA	Immune library	BoNT/A BoNT/B	–	–	neutralize both BoNT/A and BoNT/B	Neutralizing	(115)
Nb147	Immune library	S. aureus	–	–	screen for S. aureus contaminations in foods	Diagnosis	(117)
C6 C11	Immune library	SEC	–	–	detected SEC in dairy products	Diagnosis	(118)
nanobody against SEB	Immune library	SEB	–	–	detected SEB in suspicious foods	Diagnosis	(119)
L5-78 L5-79	naive library	LM	–	–	detected foodborne LM in food	Diagnosis	(120)
R303 R330 R326	naive library	LM InlB	6DBA 6DBE 6DBD	–	bound at the c-Met interaction site on InlB and preventing bacterial invasion	Neutralizing	(121)

## Single Domain Antibodies against pattern recognition receptor

Pattern recognition receptors (PRRs) are a class of receptors that play crucial roles in detecting conserved pathogen associated molecular patterns (PAMPs) shared among many microorganisms or endogenous damage-associated molecular patterns (DAMPs) to initiate downstream signaling (123–126). PRRs have been identified and are notably classified into the following families: Toll-like receptors (TLRs), the Ctype lectin receptors(CLRs), the nucleotide-binding oligomerisation (NOD)-like receptors (NLRs), the RIG-I-like receptors, the absent in melanoma 2 (AIM2)-like receptors and the OAS like receptors (127–130). PRRs connect PAMPs or DAMPs to trigger a variety of signal pathways, eventually activating interferon regulatory factor (IRFs), nuclear factor-kappa B (NF-κ B), mitogen-activated protein kinase (MAPKs) and etc., which promotes the expression of pro-inflammatory cytokines (131–133). The sdAbs against Pattern Recognition Receptor are listed in **Table 4**.

**Table 4:** Single Domain Antibody against Pattern Recognition Receptor.

Nanobody	Source	Target	Structure	(IC50)/KD	Function	Diagnosis/Neutralizing	Ref.
nanobody against TLR4	Immune library	TLR4	–	–	reduce the release of inflammatory factors and improve the survival rate of animals	Neutralizing	(134)
Nb1.46 Nb2.22	Immune library	Clec4F	7DJX 7DJY	0.2-2 nM	structural and functional investigation and as molecular imaging and therapeutic agents	Diagnosis Neutralizing	(135)

### TLR4

Toll-like receptor 4 (TLR4) is a member of the TLR family, which participates in innate immunity and mediates inflammation by recognizing lipopolysaccharide (LPS) or bacterial endotoxin (125, 136, 137). Overactivation of TLR4 can trigger the production of various inflammatory factors, which are related to the occurrence and development of a series of diseases including sepsis (138), endotoxemia, pregnancy-related disorders (139, 140), cardiovascular disease (141, 142), intestinal inflammation (143), rheumatoid arthritis (144), acute kidney injury (AKI) (145, 146), and acute lung injury (147). Therefore, the drug design and development for this target have high therapeutic potential and the anti-inflammatory effect of TLR4 inhibitors has been confirmed by several studies (148–150). Liao and his colleagues (134) identified an anti-TLR4 intermediate and C-terminal domain-recognizing nanobodies using phage display. Then, through *in vitro* and *in vivo* experiments, they confirmed that the anti-TLR4 nanobody can effectively reduce the release of inflammatory factors and improve the animal survival rate. The effect is even more pronounced when two different nanobodies are combined.

### Clec4f

C-type lectins can recognize a variety of ligands and play an important role in a variety of physiological functions. Particularly, C-type lectins contribute to innate and adaptive antibacterial immune responses by recognizing surface polysaccharides of specific pathogens (151). Clec4f is a member of the type II C-type lectin family and is only expressed by Kupffer cells (152–154). In addition, studies have shown that Clec4f is involved in α-galactose ceramide presentation and *Listeria monocytogenes* infection in mouse liver (155). Zheng et al. developed a series of nanobodies from an alpaca immunized with recombinant mouse Kupffer cell receptor Clec4F by using a phage display. After bio-panning selections, they obtained 14 different nanobodies against Clec4F with an affinity ranging from 0.2 to 2 nM. Furthermore, they have characterized the structure of two Clec4F nanobodies, Nb1.46 and Nb2.22, with different CDR2 and CDR3 sequence features. These works may contribute to the study of Clec4F structure and function as well as its use as a molecular imaging agent and therapeutic agent (135). In another study, they indicated that Clec4F nanobodies could be used to track changes in Kupffer cell (KCs) dynamics in mice *via* non-invasive imaging (153).

## Conclusion and perspectives

As bacterial antibiotic resistance is developed at increasing pace, there is a great urgency to develop a non-antibiotic approach to treat bacterial infections. SdAbs are versatile molecules with favorable properties representing an alternative tactic for both therapeutic and diagnostic applications in bacterial infections. SdAbs are characterized by minimal size, high stability, strong affinity, good solubility, and low immunogenicity which open pathways to target antigens that were previously inaccessible during bacterial infection. Therapeutic nanobodies are still in early phase development, however they have a promising future. The first therapeutic nanobody-based drug, Caplicizumab (Cablivi), was approved by EMA in August 2018 and by FDA in March 2019 for the treatment of blood clotting disorder. Since then, Ciltacabtagene autoleucel (Carvykti) a nanobody based Chimeric Antigen Receptor T cell (CAR-T)-based medication against relapsed or refractory multiple myeloma was approved by FDA and EMA (February and May, 2022) and Envafolimab, a subcutaneous injectable sdAb directed against PD-L1 (approved in November 2021) by the Chinese National Medical Products Administration (NMPA) for adult patients with microsatellite instability-high or mismatch repair deficient advanced solid tumors followed soon after. These successes demonstrate the flexibility in engineering and administration of sdAbs as well as the variety of diseases that can be tackled. It will probably not take long before sdAbs with their considerable potential as a diagnostic and therapeutic agent will enter the market for bacterial infectious diseases and will contribute to public health.

## Author contributions

QQ, HL wrote the review under the supervision of YW and SZ. WH, YG, JZ, FZ, JS and SM made the figure, tables and revised the manuscript. All authors contributed to the article and approved the submitted version.

## Funding

This work was supported by the National Natural Science Foundation of China (No. 31870132, No. 82072237), Shaanxi Province Natural Science Funding, and Institutional Foundation of the First Affiliated Hospital of Xi’an Jiaotong University. SZ was supported by Northwest A&F University Star-up Funding.

## Conflict of interest

The authors declare that the research was conducted in the absence of any commercial or financial relationships that could be construed as a potential conflict of interest.

## Publisher’s note

All claims expressed in this article are solely those of the authors and do not necessarily represent those of their affiliated organizations, or those of the publisher, the editors and the reviewers. Any product that may be evaluated in this article, or claim that may be made by its manufacturer, is not guaranteed or endorsed by the publisher.
